# Approach to diagnosis and pathological examination in bronchial Dieulafoy disease: a case series

**DOI:** 10.1186/1465-9921-9-58

**Published:** 2008-08-05

**Authors:** Antoine Parrot, Martine Antoine, Antoine Khalil, Jonathan Théodore, Gilles Mangiapan, Bernard Bazelly, Muriel Fartoukh

**Affiliations:** 1 Service de Pneumologie et Unité de Réanimation Médicale, Hôpital Tenon, Assistance Publique – Hôpitaux de Paris and Université Pierre et Marie Curie, Paris, France; 2 Service d'Anatomie Pathologique, Hôpital Tenon, Assistance Publique – Hôpitaux de Paris and Université Pierre et Marie Curie, Paris, France; 3 Service de Radiologie, Hôpital Tenon, Assistance Publique – Hôpitaux de Paris and Université Pierre et Marie Curie, Paris, France; 4 Service de Chirurgie Thoracique, Hôpital Tenon, Assistance Publique – Hôpitaux de Paris and Université Pierre et Marie Curie, Paris, France

## Abstract

**Background:**

There are limited series concerning Dieulafoy disease of the bronchus. We describe the clinical presentation of a series of 7 patients diagnosed with Dieulafoy disease of the bronchus and provide information about the pathological diagnosis approach.

**Patients and methods:**

A retrospective review of patients who underwent surgery for massive and unexplained recurrent hemoptysis in a referral center during a 11-year period.

**Results:**

Seven heavy smoker (49 pack years) patients (5 males) mean aged 54 years experienced a massive hemoptysis (350–1000 ml) unrelated to a known lung disease and frequently recurrent. Bronchial contrast extravasation was observed in 3 patients, combining both CT scan and bronchial arteriography. Efficacy of bronchial artery embolization was achieved in 40% of cases before surgery. Pathological examination demonstrated a minute defect in 3 cases and a large and dysplasic superficial bronchial artery in the submucosa in all cases.

**Conclusion:**

Dieulafoy disease should be suspected in patients with massive and unexplained episodes of recurrent hemoptysis, in order to avoid hazardous endoscopic biopsies and to alert the pathologist if surgery is performed.

## Background

Massive hemoptysis is a life threatening condition associated with a mortality rate exceeding 50% in the absence of adequate treatment [1-3]. A standardized and prompt management is required in emergency to improve survival, as well as a careful search of both the location and the etiology of bleeding. More than one hundred causes of massive hemoptysis have been described [4]. Nevertheless, no cause is identified in about 15% of the cases, despite a complete investigation including fiberoptic bronchoscopy and CT-scan [5,6].

We have recently reported a few subset of patients with so-called cryptogenic hemoptysis, in whom an unexpected vascular abnormality was demonstrated at pathological examination of the pulmonary resection, characterized by a bronchial artery running within the submucosa and called Dieulafoy disease of the bronchus [7]. Additionally, only eight cases of bronchial Dieulafoy disease proved on histological data in six case reports have been reported to date [8-13]. To our knowledge, no case series have been published from a tertiary referral center managing massive hemoptysis.

The aim of our study was to report the clinical presentation of the patients who underwent surgery for massive hemoptysis in our center and in whom a Dieulafoy disease of the bronchus was eventually diagnosed and to provide detailed information on the approach to pathological diagnosis in this field.

## Patients and methods

### Study design

All the patients were recruited in a respiratory intensive care unit of a 800-bed tertiary university hospital in Paris, France, between May 1995 and July 2006. During the study period, 810 patients were admitted to our unit for hemoptysis, 111 of whom underwent surgery. Part of those patients has already been reported from our group [7,14]. The study was conducted according to the French law which judged unnecessary ethical approval and patient consent for such a retrospective analysis of medical records.

### Diagnosis of Dieulafoy disease of the bronchus

The diagnosis of Dieulafoy disease of the bronchus was clinically suspected in case of the need for surgery for massive hemoptysis with no identifiable cause after fiberoptic bronchoscopy and CT-scan. Dieulafoy disease of the bronchus was pathologically confirmed by the evidence of an isolated and localized area of hemorrhage with no underlying lung disease known to be associated with bronchial systemic hypervascularization (such bronchiectasis) on macroscopic inspection plus the evidence of an unusual large superficial bronchial artery located into the submucosa, possibly extended through the mucosa into the bronchus lumen with no vasculitis, aneurysm or arteriosclerosis on microscopic examination.

### Pathologic lung study

The lung samples (lobectomy) were processed in an uniform way by the same pathologist (MA), when Dieulafoy disease was suspected. First, the fresh lung resection was macroscopically examined. Macroscopic inspection confirmed the focal nature of the hemorrhage. Nodular lesions, blebs, areas of induration and subpleural hypervascularization were checked. Segmental and sub segmental bronchi were subsequently opened with chisel in the absence of obvious etiology. The presence and the location of blood cloths were identified. After careful washing, bronchiectasis, bronchial inflammatory aspect and tumoral obstruction were searched as well as mucosal abnormalities, *i.*e. minute defect and ulceration. Second, after formalin fixation, 3 mm thick serial sections were performed perpendicularly to the axis of the suspected bronchus previously identified by the presence of cloths, focal areas of hemorrhage or mucosal abnormalities; the same procedure was applied to the other bronchi for comparison. Samples of fixed tissue were processed into paraffin block, perpendicularly to the bronchial axes. Third, serial sections were performed on the paraffin block focusing on the suspected bronchus until lesions were found. Sections were stained with hematoxylin-eosin-safran, and elastic stain (Miller stain) for vessel identification.

### Collection of patients' data

The following prospectively collected clinical data were extracted from our data base and controlled with the review of the medical charts: baseline demographics, drug intake, comorbid conditions, severity of hemoptysis, clinical presentation, laboratory tests, chest radiography, fiberoptic bronchoscopy and CT scan, ICU management and vital status at ICU discharge.

## Results

Seven patients fulfilled the aforementioned criteria during the 11-year study period.

### Patients' characteristics

The patients (5 males) were aged 54.3 ± 11.5 years (range, 38 to 69 years). They were current heavy smokers (49 ± 28.5 packs/years); all but one were alcohol abusers. Two had mild to moderate chronic obstructive pulmonary disease. One patient had a history of pulmonary tuberculosis. Two patients were treated for systemic hypertension, 1 of whom had a history of a transient ischemic stroke and unexplained intestinal bleeding (Additional file 1). A previous episode of hemoptysis had occurred in 5 patients, 3 of whom had previously received a bronchial artery embolization (BAE). Of note, 1 patient (patient n°4) had been managed in our center 19 months ago for a first episode of massive hemoptysis (amount of 400 ml). Four fiberoptic bronchoscopies were necessary to locate the bleeding in the left upper lobe, as CT scan showed bilateral ground glasses. A successful BAE was performed. The patient refused a secondary scheduled surgery despite the staff decision.

The cumulative amount of bleeding ranged from 350 ml to more than 1000 ml on admission to our unit. There were, however, mild clinical and biological consequences of the bleeding. Bedside chest X-ray was unremarkable. High resolution (n = 5) or multidetector (n = 2) CT-scan angiography showed ground glass opacities in all patients, that were isolated or associated with alveolar opacities (n = 4). The upper lobes were mainly involved (n = 5). Of note, a frank contrast media extravasation within the lumen of the bronchus related to the focal hemorrhagic area was evidenced using multidetector CT-scan angiography (patient n°4). There was no lung parenchyma abnormality suggestive of carcinoma, bronchiectasis or tuberculosis. A flexible fiberoptic bronchoscopy was performed within the first 24 hours of admission, to locate both the side and site of the bleeding. Overall, 10 bronchoscopic procedures were performed. A bilateral bronchial flooding by blood was evidenced in all patients. The location of the bleeding was successfully performed after bronchoscopic techniques in 5 patients.

### Management

**Figure 1:**
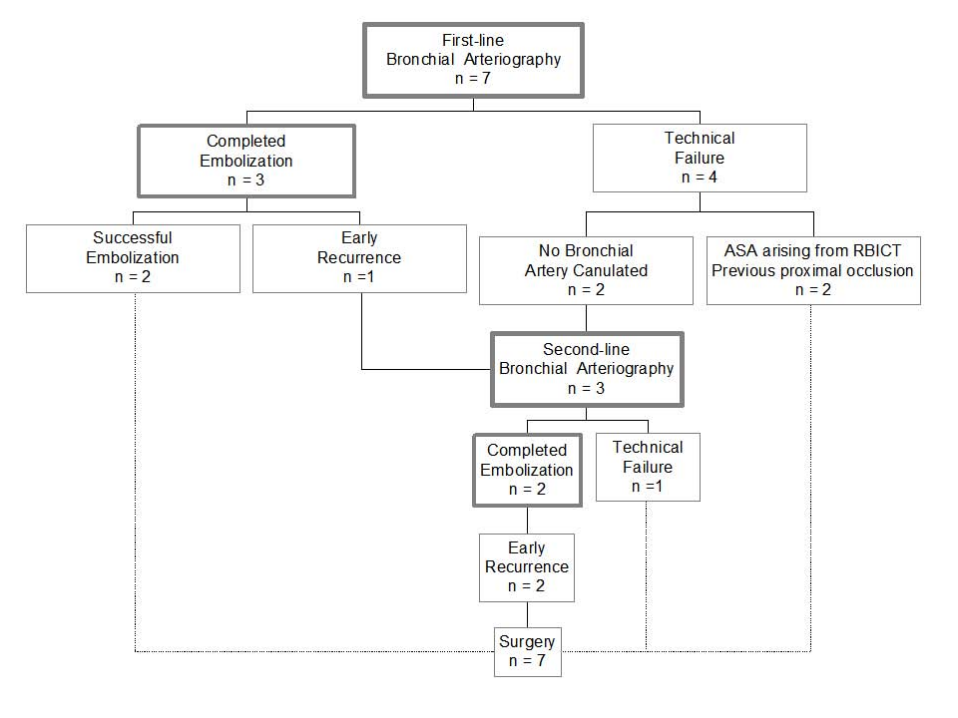
**Bronchial arteriography efficacy**. ASA = anterior spinal artery; RBICT = right broncho-intercostal trunk.

The therapeutic management was standardized, as described elsewhere [14]. Bronchoscopic techniques were attempted to control the bleeding, combining blood aspiration and local instillation of cold saline lavage. Vasoconstrictive agents were delivered bronchoscopically (adrenalin, n = 4), intravenously (terlipressin, n = 1) or both (n = 1). A BAE was first attempted in all patients totaling 10 sessions before surgery (Figure 1). Technical failure of bronchial arteriography was related to failure of canulation in 2 patients (patients n°1 & n°3) and anatomical consideration in 2 others (patients n°5 & n°6). All bronchial arteries draining the bleeding site were enlarged without systemic to pulmonary artery shunting. A frank contrast media extravasation into the bronchial lumen was evidenced in 2 patients (patients n°2 & n°7). Altogether, BAE was completed in 5 cases (4 patients) and controlled the bleeding in 2 cases (2 patients). According to the high initial amounts of bleeding, all the patients were secondary referred for surgery after 6.7 ± 5.8 days (median time 5 days). The lobe to remove, from which the bleeding originated, was identified by the combination of clinical examination, chest X-ray, CT scan and bronchoscopic findings. Bronchial arteriography was not used for locating the bleeding.

### Follow-up after surgery

All patients were alive at hospital discharge. Follow-up data were available for a mean duration of 35 ± 30 months (range, 6 to 96 months) after surgery. All but one patient (patient n°4) remained free of bleeding recurrence during follow-up. This latter patient was treated successfully with BAE. The patient n°7 died from an ischemic stroke 12 months later.

### Pathological findings

**Figure 2:**
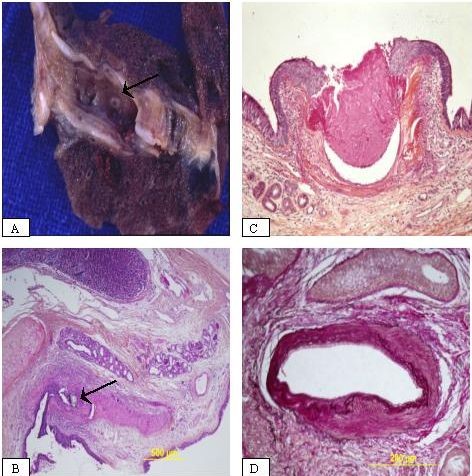
**Pathological Findings**. Macroscopic view showing a minute defect within the bronchial tree (arrow) (A). Low-power view showing the location of the vessel in the sub-mucosa beneath the cartilage plate, and the presence of the material of embolization in the lumen (arrow) (B). High-power view revealing the protrusion of the superficial vessel in the lumen with an ulceration and a squamous metaplasia of the epithelium (C). High power-view showing a dysplastic artery with elastic stain (Miller stain) (D).

Lung macroscopic examination located the pathological area in all patients by showing a focal hemorrhagic area within the parenchyma and identifying a localized clot in the corresponding segmental (n = 5) or sub segmental bronchus (n = 2) (Additional file 2). Moreover, a minute bronchial mucosal defect was eventually observed (n = 3) (Figure 2A). There was no evidence of bronchial or vascular chronic disease. The macroscopic inspection was unremarkable, except blebs (patients n°1 & n°2) and nodular mass (patient n°6) that were distant from the pathological hemorrhagic area.

Microscopic examination revealed a large and dysplasic superficial bronchial artery in the submucosa in all patients beneath the cartilage (Figure 2B). Artery ulceration with rupture into the bronchial lumen was observed in 3 patients (Figure 2C). The structure of the bronchial artery appeared dysplastic with irregular thickness of the wall, which was either fibrotic or rich in elastic fibrils and had a tortuous appearance when cut at various angles. These arteries appeared on Miller stain with a thick internal lamina and a thin external one, in favor of the bronchial nature of the artery (Figure 2D). Finally, we observed (patient n°4) the material of embolization in the lumen of the vessel in 1 patient (Figure 2B). The respiratory epithelium was either clearly or slightly eroded and appeared sometimes metaplastic, although the structure of the bronchus remained normal. Of note, the nodular mass aforementioned (patient n°6) was related to a granulomatous disease consistent with an old sarcoidosis. In another patient (patient n°1) a deposit of amyloidal structure was identified. Otherwise, histological examination was unremarkable. No evidence of chronic bronchial or other vascular disease was identified. No microorganisms were grown, after special staining and culture for mycobacteria and fungi.

## Discussion

Our study aimed at better describing the process for diagnosing the Dieulafoy disease of the bronchus, from the clinical suspicion to the pathological confirmation, based on a series of 7 patients who underwent surgery for massive hemoptysis in a referral center over a 11-year period. The condition was clinically suspected in heavy smokers with recurrent and unexplained episodes of massive hemoptysis, characterized by amounts of bleeding both high and rather disproportioned, as compared with those usually reported in patients presumed to have a cryptogenic hemoptysis. Although there were no specific CT-scan or angiographic criteria, the frequent findings of both the direct and frank contrast media extravasation within the suspected bronchial lumen and the enlarged aspect of the bronchial artery without systemic to pulmonary artery shunting were suggestive of the vascular anomaly. A subsequent structured and rigorous pathological examination of the surgical lung resection confirmed definitively the diagnosis.

Dieulafoy disease is a vascular anomaly characterized by the presence of a dysplastic artery in the submucosa. It was first reported in the gastrointestinal tract, accounting for up to 2% of the bleedings [15]. The disease has been recently described in the respiratory tract [12]. However, the incidence of the Dieulafoy disease of the bronchus is unknown and probably slightly underestimated regarding to the rigorous pathological procedure needed for establishing the diagnosis. In our experience, Dieulafoy disease of the bronchus accounted for at least 6% of the patients undergoing surgery for hemoptysis overall and up to 55% of the patients undergoing surgery for hemoptysis presumed to be cryptogenic [7].

The diagnosis of Dieulafoy disease of the bronchus should be suspected on the combination of history, clinical features and imaging investigations in order to consider surgical treatment and alert the lung pathologist. First, our patients were heavy smokers, similarly to the previous published isolated case reports. Second, respiratory past history was unremarkable, except unexplained and severe episodes of hemoptysis in 5 patients, 3 of whom had received a BAE (Additional file 1). Third, hemoptysis was massive and presumed to be cryptogenic, since no cause was identified after physical examination, fiberoptic bronchoscopy and CT-scan. Conversely to the gastrointestinal disease for which the endoscopic findings are diagnostic, we did not use bronchoscopic criteria to diagnose the vascular disease because the source of bleeding may be difficult to assess during active massive hemoptysis [16-18], the bronchial abnormalities may be sub segmental and therefore not accessible and the small size of the bronchial lesion (usually less than 10 mm) may be difficult to detect when surrounded by clots. However, a few mucosal abnormalities have been bronchoscopically described in this setting, such as a smooth elevated non pulsating lesion [13] or a nodular lesion within a normal overlying mucosa [9]. It should be emphasized that these later bronchoscopic findings are not specific and may be related to bronchial artery aneurysms, arteriovenous malformations or small cancers [19-21]. As the amount of bleeding related to Dieulafoy disease may be massive, bronchial biopsies should be avoided in this setting, even during a period of non active bleeding [9,13]. Moreover, in our opinion, performing biopsy should be not useful in this setting, since the diagnosis of Dieulafoy disease of the bronchus should be based on the pathological examination of a large surgical lung resection. Fourth, the bronchial arteriography findings did neither evidence systemic to pulmonary shunts nor aneurysms. Additionally, the arteries appeared all enlarged and frank contrast media extravasation in the bronchial lumen was frequent, when combining the findings of both BAE (n = 2) and multidetector CT-scan angiography (n = 1). These findings are in accordance with those reported by *Durham et al *in gastrointestinal Dieulafoy disease [22]. Conversely, little angiographic data are available regarding to the bronchial artery disease [8-10,12,23,24]. Dilated vessels have been described [9,10,23], especially in association with specific parenchymal diseases [10,23]. Last, although no firm conclusions can be drawn regarding to the small size of our population, our study highlights the poor efficacy of BAE in this clinical setting, as compared with the usual 80% to 90% successful rate of bleeding control using this procedure [17]. Nevertheless, owing to the morbidity and the mortality related to emergency surgery performed during active bleeding, we recommend to attempt BAE as the first-line therapeutic approach [7]. Additionally, some patients with a non-diagnosed Dieulafoy disease may have probably been treated with BAE.

To our knowledge, our series is the first to carefully describe the pathological investigation to diagnose the Dieulafoy disease of the bronchus on surgical lung resection. The main pathological criterion is the evidence of a large and superficial bronchial artery located within the sub mucosa [8-13]. In our series, the macroscopic analysis was crucial to detect a minute mucosal defect, as usually observed in the Dieulafoy disease of the gastrointestinal tract. As ectopic bronchial arteries have also been described during chronic pulmonary diseases [17,25,26], a special attention was made to exclude chronic lung diseases, such bronchiectasis and other inflammatory processes or carcinoma. Additionally, CT scan demonstrated no parenchymal abnormalities, except ground glass or alveolar opacities reflecting the severity of bleeding [27]. Although a few parenchymal abnormalities (blebs, nodular mass and amyloidal deposit) were pathologically evidenced, those later were actually distant from the focal hemorrhagic area and consequently not considered as the cause of bleeding.

The pathogenesis of Dieulafoy disease remains unclear. Whether the origin of the anomaly is congenital, acquired or merely a variation of normal is not known. As suggested in our study, age and/or tobacco use may be predisposing states to the occurrence of the disease. Even if the dysplastic artery wall change may contribute to its weakness, the trigger factor of the vessel rupture is not known. Does the vessel rupture occur after a mucosal injury or does the vessel pressure induce a mucosal defect is unclear. Furthermore, the nature of the vessel that is bleeding remains controversial. Some have suggested that the artery belongs to the pulmonary vasculature owing to the failure of BAE, while others identified the vessel as originating from the systemic vasculature [10,13]. In our series, the angiographic data are supporting this latter hypothesis, as well as the pathological findings of the vessel with elastic stain and the intravascular evidence of material of embolization (patient n°4) (Figure 3B). Last, Dieulafoy disease might be a part of a general disease as suggested by the clinical history and outcome of the patient n°4.

The limitations of our study are related to its retrospective nature and to the fact that it was conducted on patients undergoing surgery in a referral center with an extensive experience of severe hemoptysis.

In summary, Dieulafoy disease of the bronchus should be considered in a heavy smoker patient with unexplained and recurrent massive hemoptysis, after a rigorous confrontation of both clinical and radiological findings. Useless and dangerous bronchial biopsies should be avoided. The therapeutic approach should be surgical and the pathological examination should be structured and meticulous.

## Abbreviations

BAE: Bronchial artery embolization.

## Competing interests

The authors declare that they have no competing interests.

## Financial support

None

## Authors' contributions

AP had full access to the data and takes responsibility for the integrity of the data at the accuracy of the data analysis. MA performed the pathological analysis. All authors read and approved the final manuscript.

## Supplementary Material

Additional file 1 **Table S1.** Patients' characteristics, management and outcome. Comparison with the literature cases. Click here for file

Additional file 2 **Table 2.** Pathological findings. Comparison with the literature cases. Click here for file
