# Supplementary material for: Approach to diagnosis and pathological examination in bronchial Dieulafoy disease: a case series
Source: Respir Res. 2008 Aug 5;9(1):58. doi: 10.1186/1465-9921-9-58 (PMC2529287; doi:10.1186/1465-9921-9-58)
Supplement: Additional file 1 — Table S1. Patients' characteristics, management and outcome. Comparison with the literature cases. [file 1465-9921-9-58-S1.doc]

# Table 1.

| Patient | Sex | Age,  years | Tobacco  pack year | History of hemoptysis | Respiratory  history | Extra respiratory history | Bleeding amount, mL | Fist-line treatment | Vital status | Follow-up,  months |
| --- | --- | --- | --- | --- | --- | --- | --- | --- | --- | --- |
| Our series | | | | | | | | | | |
| n°1 | M | 69 | 60 | Yes | COPD | Hypertension | 500 | BAE | Alive | 31 |
| n°2 | M | 45 | 60 | Yes | No | No | 1000 | BAE | Alive | 49 |
| n°3 | M | 54 | 30 | No | CB | No | 1000 | BAE | Alive | 96 |
| n°4 | F | 57 | 50 | Yes | Tuberculosis | Renal Aneurysm | 600 | BAE | Alive | 28 |
| n°5 | M | 49 | 30 | No | No | Epigastric Pain | 350 | BAE | Alive | 25 |
| n°6 | M | 38 | 13 | Yes | No | No | 500 | BAE | Alive | 6 |
| n°7 | F | 68 | 100 | Yes | No | Hypertension  Stroke  Intestinal Bleeding | 800 | BAE | Dead | 12 |
| Literature | | | | | | | | | | |
| n°8  (Sweerts et al ^12^) | F | 35 | 0 | Yes | No | No | Massive | Bronchoscopy | Alive | 4 |
| n°9  (Sweerts et al ^12^) | M | 59 | 40* | No | No | No | NA | Surgery | Alive | NA |
| n°10  (Kuzucu et al ^8^) | M | 28 | 15 | Yes | Tuberculosis | No | Massive** | Surgery | Alive | 12 |
| n°11  (Kuzucu et al ^8^) | M | 45 | 35 | Yes | No | No | Massive | BAE | Alive | 5 |
| n°12  (VanderWerf et al^13^) | F | 70 | 60 | Yes | Tuberculosis  COPD | No | Massive** |  | Dead |  |
| n°13  (Stoopen et al ^11^) | M | 51 | 40 | No | No | Epistaxis | 500 | Surgery | NA | NA |
| n°14  (Pomplun et al ^10^) | M | 32 | NA | No | No | No | Massive | BAE | NA | NA |
| n°15  (Loschhorn et al ^9^) | F | 47 | NA | No | No | No | 500** | Surgery | Alive | 72 |

M= male; F=female; COPD= chronic obstructive pulmonary disease; CB=chronic bronchitis; NA= not available; BAE= bronchial artery embolization.

*fourty cigarette per day; ** Hemoptysis post biopsy.
