# Supplementary material for: Approach to diagnosis and pathological examination in bronchial Dieulafoy disease: a case series
Source: Respir Res. 2008 Aug 5;9(1):58. doi: 10.1186/1465-9921-9-58 (PMC2529287; doi:10.1186/1465-9921-9-58)
Supplement: Additional file 2 — Table 2. Pathological findings. Comparison with the literature cases. [file 1465-9921-9-58-S2.doc]

# Table 2.

| Patient | Macroscopic  examination | | | | Microscopic  examination | | | | |
| --- | --- | --- | --- | --- | --- | --- | --- | --- | --- |
|  | Location | Focal  hemorrhagic area | Bronchial  clot | Minute  defect | Superficial  artery | Dysplatic  artery | Artery  ulceration | Bronchial  ulceration | Associated  lesions |
| Our Series | | | | | | | | | |
| n°1 | RUL | + | + | + | + | + | - | + | Blebs  Amyloidal deposit |
| n°2 | RUL | + | + | + | + | + | + | + | Blebs |
| n°3 | LLL | + | + | + | + | + | - | + |  |
| n°4 | LUL | + | + | - | + | + | + | + |  |
| n°5 | LUL | + | + | - | + | + | - | + |  |
| n°6 | RLL | + | + | - | + | + | + | + | Nodular mass |
| n°7 | RUL | + | + | - | + | + | - | - |  |
| Literature | | | | | | | | | |
| n°8  (Sweerts et al ^12^) | RLL | NA | NA | NA | + | NA | + | + |  |
| n°9  (Sweerts et al ^12^) | RLL | NA | NA | NA | + | NA | NA | NA |  |
| n°10  (Kuzucu et al ^8^) | LLL | NA | NA | NA | + | + | + | + | Foreign body with giant cells |
| n°11  (Kuzucu et al ^8^) | ML | + | + | - | + | + | NA | NA | BOOP |
| n°12  (VanderWerf et al^13)^ | LUL | NA | NA | NA | +* | NA |  | + | COPD |
| n°13  (Stoopen et al ^11^) | ML & RLL | + | - | - | + | + | + | + |  |
| n°14  (Pomplun et al ^10^) | RUL | + | + | NA | +* | + | + | + |  |
| n°15  (Loschhorn et al ^9^) | ML | NA | NA | NA | + | + | - | - |  |

RUL = right upper lobe; RLL = right lower lobe; ML = middle lobe; LUL = Left upper lobe; LLL = left lower lobe; + = present; - = absent; NA = not available; BOOP = bronchiolitis obliterans organizing pneumonia; COPD = chronic obstructive pulmonary disease. * Pulmonary artery branch presumed to be the involved with the vascular anomaly
